# Molecular docking reveals Chitosan nanoparticle protection mechanism for dentin against Collagen-binding bacteria

**DOI:** 10.1007/s10856-022-06665-4

**Published:** 2022-05-13

**Authors:** Ziliang Zhou, Yanyan Yang, Lu He, Junmei Wang, Jie Xiong

**Affiliations:** 1 Department of Oral Emergency and General Dentistry, Affiliated Stomatology Hospital of Guangzhou Medical University, Guangdong Engineering Research Center of Oral Restoration and Reconstruction, Guangzhou Key Laboratory of Basic and Applied Research of Oral Regenerative Medicine, Guangzhou, China; 2 Beijing Stomatological Hospital, Capital Medical University, Beijing, China

## Abstract

The medical application of chitosan (Cs) has been for about half a century, but the molecular mechanism has not been elucidated yet. This study is to explore the antibacterial mechanism of chitosan nanoparticles (Csnp) in dentin at the atomic resolution level. Extracted tooth specimen was prepared in three groups: A. control group; B. Csnp treatment under ultrasonic agitation (UA); C. Csnp treatment without UA. A scanning electron microscope (SEM) was used to observe the Csnp distribution on the dentin surface. The incubations of *Enterococcus faecalis* (*E. faecalis*) were performed. Further, we explored the protection mechanism of chitosan polymers to collagen type I, using molecular docking technique and crystal structure superimposition analysis. We revealed that Csnp under UA was evenly distributed on the dental surface and the Csnp-pretreated dentin had great antibacterial activity for *E. faecalis*. Our work demonstrated that Csnp occupied the grooves of the triple-helical collagen surface, strengthened by crosslinking, and interfered with the bond of collagen adhesin through steric hindrance effect and interrupting hydrophobic interaction. Csnp protects dentin against *E. faecalis* by interacting and crosslinking with collagen type I and prevents bacterial collagen adhesin binding through steric hindrance effect and interrupting hydrophobic interaction.

**Figure:**
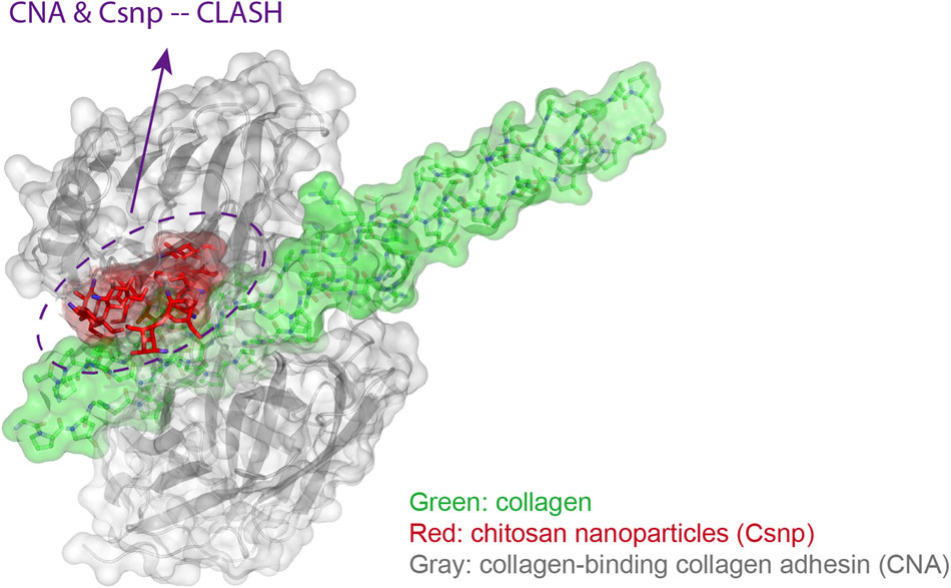
Graphical abstract

Graphical abstract

## Introduction

Chitosan, poly[β-(1,4)-2-amino-2-deoxy-D-glucopyranose], was firstly synthesized through deacetylation of polysaccharide chitin by the French physiologist Charles Rouget in 1859 [1]. Chitosan is insoluble in water or most organic solvents, but it can be dissolved in dilute acid, depending on the degree of deacetylation (DD) and the chain length [2]. Chitosan has excellent biological properties, including remarkable biosafety, mucosal adhesion, blood compatibility, and biodegradability. Moreover, chitosan has the properties of anti-tumor, antioxidant, and antibacterial [3]. Because of its outstanding biocompatibility and excellent performance, chitosan has been widely used in various fields such as pharmaceutical drug delivery, tissue engineering, implants, genetic engineering, vaccine adjuvants, and wound healing [4–6]. Chitosan is also widely used in the prevention and treatment of various oral diseases because of its excellent biological properties, such as antibacterial properties, drug-loading function, remineralization, and osteogenesis [7–10].

Chitosan has a wide range of antibacterial activities against oral bacteria and fungi, mainly relying on the cell surface. Chitosan can also trigger intracellular antibacterial effects on different microbial species [11, 12]. The adhesion and reproduction of bacteria on the inner wall of the root canal are the key factors affecting the curative effect of root canal therapy and post-core adhesion. *E. faecalis* is the primary pathogen of root canal infection. Chitosan showed significant antibacterial properties against *E. faecalis* both in floating state and biofilm formation through charge interaction [13]. Previous studies have shown that bacteria cells are strongly affected by polycationic Csnp because of the large surface area and the high charge density [14]. There are four accepted hypotheses of chitosan activities on Gram-positive and -negative bacteria: the polycationic chitosan causes the release of intercellular components; binding to bacterial DNA for mRNA inhibition; blocking the nutrient flow; preventing the chelation of essential metals [15]. To date, there is still no consensus on the mechanism of chitosan antimicrobial activity, which defers the applications and further development.

Our previous studies have confirmed that Csnp binding to dentin in combination with 1-Ethyl-3-(3-dimethyl aminopropyl)-carbodiimide (EDC) and N-hydroxysuccinimide (NHS) reduced the degradation of dentinal collagen [16]. The UA created a uniform distribution of Csnp on the etched dentin surface. Csnp infiltrated and bound onto mostly exposed collagen networks, even in dentinal tubules [17]. In this study, we pretreated the dentin with EDC-crosslinking Csnp under UA, observed the Csnp distribution on the dentin surface by SEM, and then examined the bacterial adhesion to the Csnp-binding dentin surface using colony-forming unit (CFU) assay. Further, we explored the molecular mechanism through molecular docking technique and crystal structure analysis, hoping to answer why the EDC-crosslinking Csnp has antibacterial properties to *E. faecalis* at the molecular level. This is of great significance to the target engineer, chemical modification, and performance improvement of chitosan application for future studies.

## Materials and methods

### Csnp preparation

Csnp (5.0 mg/ml) was freshly prepared by the ionic gelation method as previously described [18, 19]. Cs (Sigma, St.Louis, USA) was dissolved in acetic acid and purified twice. The Cs solution was filtered. Cs was precipitated by adding sodium hydroxide solution and dried in a vacuum dryer at 40 °C for 24 h. Crosslinking agent EDC/NHS were synthesized and prepared in the same way described in a previous paper [16].

### Specimen preparation

The inclusion criteria of the twenty-one extracted human teeth were caries-free, single-rooted teeth with similar radicular length, which mostly were maxillary incisors and single-rooted premolars. Roots with cracks or fractures were excluded. The teeth were randomly selected from a collection of human teeth and no patient identifiers were associated with the teeth.

The extracted teeth were stored in saline at 4 °C and were used within three months after extraction. The teeth were sectioned transversely near the cementoenamel junction (CEJ) using a low-speed diamond saw (Isomet, Buehler, Lake Bluff, IL, USA). The working length was set with a size #10 K file (Mani, Tochigi, Japan) at 1 mm short from the apex, and the root canals were shaped using Ni-Ti rotary instruments Protaper (Dentsply-Maillefer, Ballaigues, Switzerland) to F3 (#30, 0.30 mm). All root canals were irrigated with 2.5% sodium hypochlorite and 17% EDTA solutions alternately when the drills were changed, then rinsed with plenty of saline solution. The apical foramina were sealed with resin cement, and the post space was formed to #4 with Macro-lock drills (RTD, St Égrève, France) to a depth of 8 mm from the CEJ. Then the roots were stored in deionized water at 4 °C for further usage.

The twenty-one roots were treated in different protocols (*n* = 7) according to groupings after etching. Group A: control group (no treatment); group B: treated with Csnp solution under ultrasonic agitation for 60 s and immersed in EDC-crosslinking solution for 24 h; group C: static immersion for 24 h in Csnp and EDC-crosslinking solution for 24 h.

### Microscopy observation

SEM was used to observe the Csnp incorporated onto the dentin surface and infiltrated into the dentinal tubules. After treatment, one root was randomly selected from each group, split longitudinally, fixed overnight in 2.5% glutaraldehyde, and washed with 0.1 M phosphate buffer. The specimens were serially dehydrated using ethanol, sputter coated with palladium, and examined using SEM at magnifications of 5000. Each sample was imaged at standard sites along the root canal.

### Colony-forming unit (CFU) assay

The inoculum was prepared from a single colony of *E. faecalis* (ATCC 29212) incubated in BHI broth at 37 °C for 14 h and was adjusted to 1 × 10^6^ cells/ml. Eighteen roots after treatments were placed respectively into an Eppendorf tube with 1 ml BHI, and then 200 μl of *E. faecalis* inoculum was inoculated in each tube at 37 °C for 24 h. The specimens were split into two halves and rinsed gently to remove loose non-adherent cells. The dentinal shavings at a depth of 1 mm were collected using a bur (three sites), transferred to 1 ml BHI in an Eppendorf tube, and incubated at 37 °C for 4 h to enrich the bacterial cells before plating. The samples were serially diluted in BHI. Then, 10 μl Aliquots were plated onto BHI plates and incubated at 37 °C for 24 h. The bacterial colonies were counted, and the CFU value was transformed into a log CFU value.

### Molecular docking studies

In order to explore the basis of the EDC-crosslinking initiation, docking studies were performed between a synthetic collagen peptide and different lengths of chitosan polymers, including 3mers, 6mers, 9mers, and 12mers. Chitosan oligomers were set as the models for Csnp here, owing to the violently growing probabilities of the flexible structure simulation as chitosan polymers extended. The model of the synthetic collagen peptide was obtained from the crystal structure of *Staphylococcus aureus* (*S. aureus*) collagen-binding collagen adhesin (CNA) in complex with the collagen peptide (PDB: 2F6A) [20]. The chitosan polymers were generated by ChemDraw Professional 16.0 and converted to 3D models by iBabel 5.0 [21]. After the coordinate preparation, the collagen peptide was set as the macromolecule, while the chitosan polymers were the ligands. Using AutoDock Vina [22], the center of the grid box was chosen as the coordinate (6, −5, 30), and the size of the grid box was set as 40 × 24 × 25. The grid box included half of the whole collagen peptide, which was symmetric to the other half. The coordinate of the collagen was fixed, and the chitosan polymers flexibly dock to the collagen, respectively. The docking results were visualized by using PyMOL 2.5.1 [23].

### Crosslinking sites evaluation

Human collagen type I is composed of two α1 chains and one α2 chain. The sequences of the two human collagen type I chains were fetched from the Uniprot website (https://www.uniprot.org). The sequences were analyzed manually. The content of the acidic amino acids (glutamic acid and aspartic acid) was calculated for evaluating EDC-crosslinking reactions.

### Structure and docking model analysis

Since the chitosan polymers have docked to the synthetic collagen peptide from the co-crystal structure (PDB: 2F6A), the results of flexible docking superimposed on the CNA and collagen complex structure. All the best docking results of the chitosan polymers have been analyzed, including the steric hindrance effect and the hydrophobic interaction interference.

## Results

### Csnp distribution on the dentin surface

**Fig. 1:**
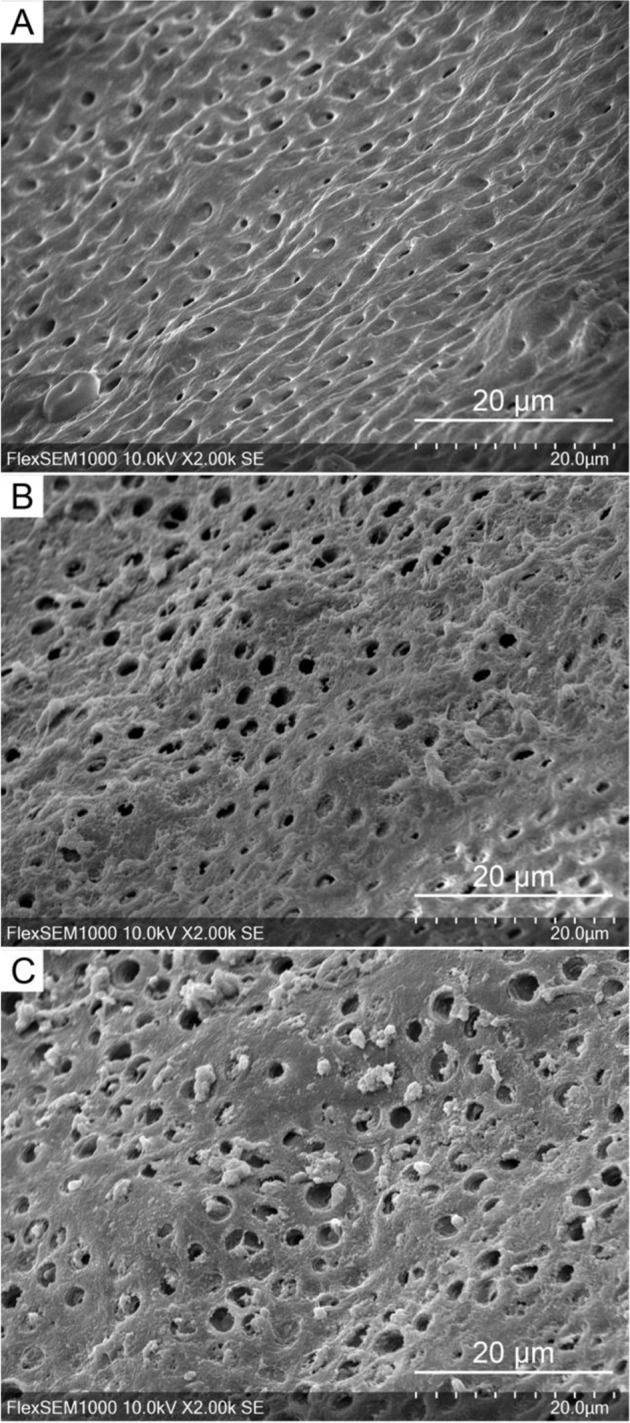
Scanning electron micrographs of Csnp on dentin surface: no treatment, 2000× (**A**); Csnp with EDC under UA, 2000× (**B**); Csnp with EDC on static immersion, 2000× (**C**)

The SEM images showed different dentin surfaces in groups. In Fig. 1A, etched root canal dentin in the control group varied at different levels of collagen exposure because of different demineralization extent. Collagen was more exposed at some dentin sites, while the other dentin was barely demineralized without collagen exposed. Csnp was much more observed on the dentin surface in group B (Fig. 1B) than in group C (Fig. 1C). Under UA, Csnp was distributed evenly in the canal wall and dentinal tubule orifices. Some Csnp even infiltrated in the dentinal tubules, especially in the areas with collagen partially exposed (Fig. 1B). In group C, Csnp aggregated around the tubular orifice and scattered sparsely (Fig. 1C).

### Analysis of bacterial reproduction

CFU differed among groups. Group B and C, as the experimental groups, both showed a significant reduction of bacteria (*P* < 0.05). Group B displayed the lowest CFU value under UA. CFU was converted into Log CFU then. Log CFU in group B was the lowest among all the groups, statistically lower than groups A and C.

### Chitosan polymers bind to the collagen peptide

**Fig. 2:**
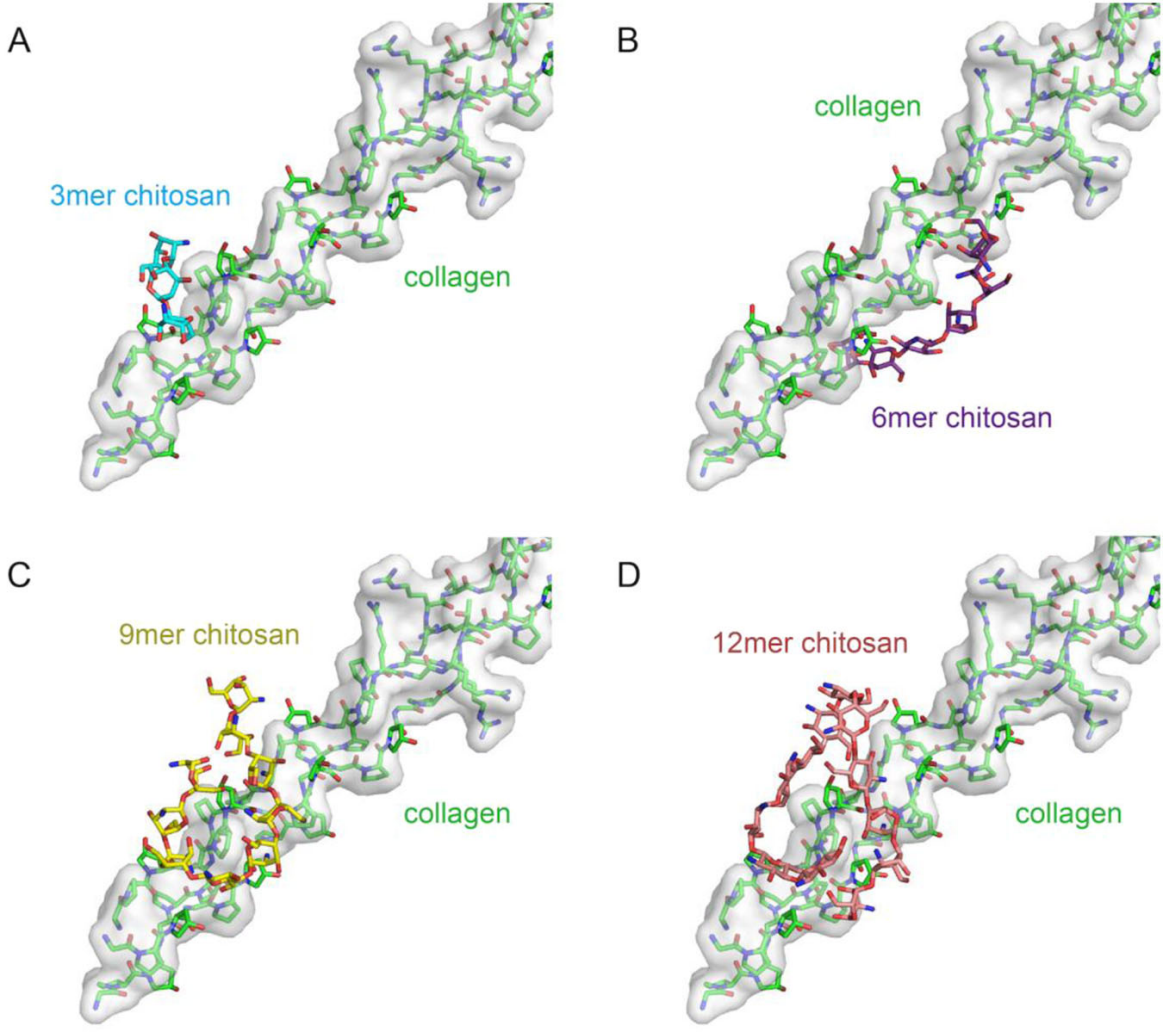
The best docking results of the chitosan with 3mer (**A**), 6mer (**B**), 9mer (**C**), and 12mer (**D**) to the synthetic collagen peptide (PDB code:2F6A). The synthetic collagen peptide is colored green and shown as a transparency surface model. The chitosan polymers are colored cyan (3mer), purple (6mer), yellow (9mer), and pink (12mer), respectively

The AutoDock Vina revealed that the synthetic collagen peptide (PDB: 2F6A) had potential binding sites for chitosan with 3mers, 6mers, 9mers, and 12mers (Fig. 2), having different affinities of 2.28 mM, 590 μM, 1.16 mM, and 1.37 mM, respectively. Although the affinities were not high enough for stable binding, they were sufficient for initiating EDC-crosslinking reactions.

**Fig. 3:**
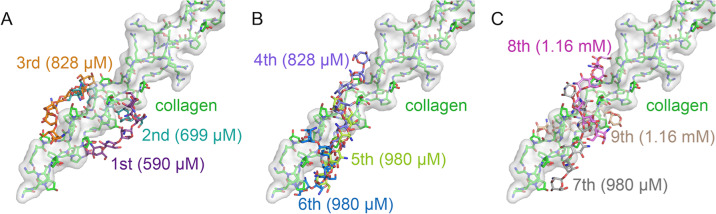
The best nine docking results of 6mer chitosan to the synthetic collagen peptide (PDB code:2F6A) with slightly different affinity, nominating in descending order of the predictive binding affinity (KD), as 1st to 3rd (**A**), 4th to 6th (**B**), and 7th to 9th (**C**). The synthetic collagen peptide is colored green and shown as a transparency surface model

According to the docking results, we found that the chitosan molecules shaped from lines (3mers and 6mers) gradually to balls (9mers and 12mers) as the chitosan polymers extended (Fig. 2). All the chitosan polymers bound to the collagen by occupying the grooves circling the surface of the collagen triple helix. Therefore, we indicated that the collagen surface had various binding sites for chitosan polymers, the structural basis for subsequent EDC-crosslinking (Fig. 3).

### Human collagen type I has abundant carboxyl groups for crosslinking with chitosan

**Fig. 4:**
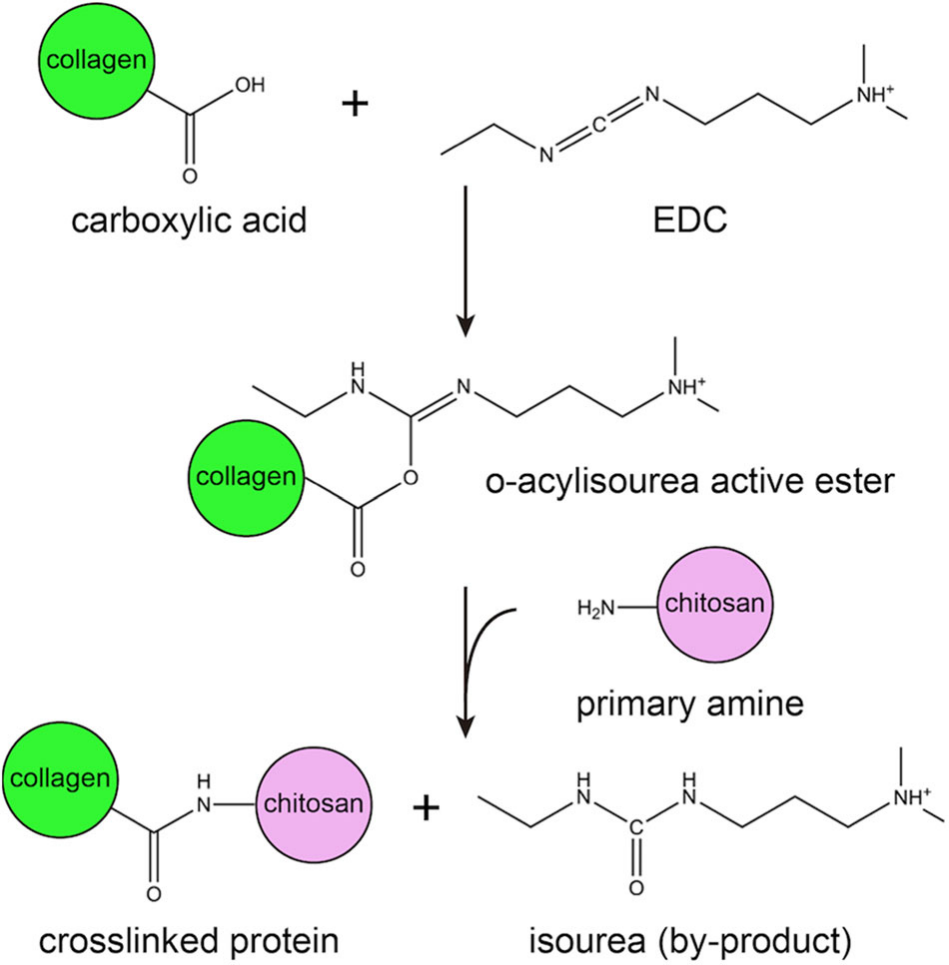
EDC-crosslinked reaction scheme between human collagen type I and chitosan

Soluble EDC is a crosslinking agent that catalyzes amide bonds between the carboxyl and primary amine groups via the formation of O-acylisourea derivatives. The addition of NHS can increase the efficiency of EDC-crosslinking reactions (Fig. 4) [24]. In other words, the acidic amino acids (glutamic acid and aspartic acid) of human collagen type I provide the carboxyl groups, while the chitosan contains the primary amine groups (Fig. 4), for amide bond formation.

Human collagen type I is a heterotrimer [25] composed of two α1 chains (UniProt: P02452) and one α2 chain (UniProt: P08123). The α1 chain comprises 1464 amino acids with 66 aspartic acids and 75 glutamic acids, while the α2 chain is composed of 1366 amino acids with 43 aspartic acids and 66 glutamic acids. The acidic amino acid ratio of the α1 chain is 9.63%, and the ratio of the α2 chain is 7.78%. Therefore, the overall acidic amino acid ratio of the human collagen type I molecule is 9.08%, not including the C-terminal carboxyl groups of the three chains. In the collagen structures, all the side chains of the amino acids are toward outside from the center of the linear triple-helical molecule. This means that human collagen type I provides sufficient (9.08%) amino acid sites for chitosan crosslinking.

### chitosan prevents bacteria binding to collagen type I

The previous study of the co-crystal structure has shown that *S. aureus* CNA, a 135 kDa cell wall-anchored protein, is in complex with a synthetic collagen-like triple-helical peptide [20]. Since then, homologous proteins were identified in other Gram-positive bacteria, including ACE (adhesin of collagen from enterococci) in *E. faecalis* with the affinity of 48 μM to collagen type I [26, 27]. The ACE structure is very similar to *S. aureus* CNA and binds to collagen by a similar ‘Collagen Hug’ mechanism [28]. Thus, the co-crystal structure analysis of the CNA-collagen complex can represent the ACE binding mechanism in this study.

**Fig. 5:**
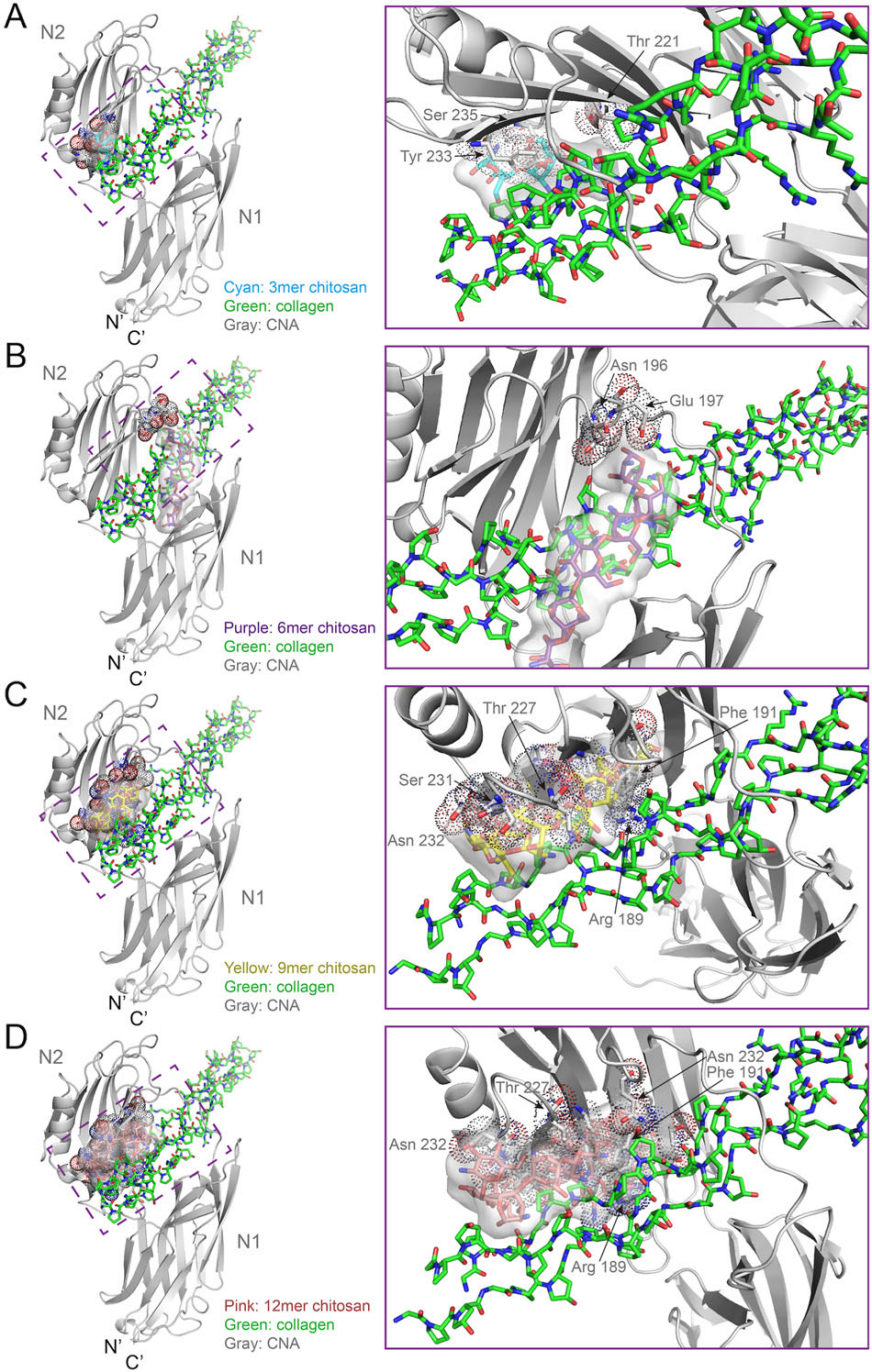
The CNA N1 domain clashes to the best binding models of chitosan polymers with 3mer (**A**), 6mer (**B**), 9mer (**C**), or 12mer (**D**), respectively. Views of the detail clashes are shown in the right frames. N′ and C′ represent the N termini and the C termini of the CNA protein. The feature residues are shown as ball-and-stick and dot models. The chitosan polymers are shown as ball-and-stick and transparency surface models, colored cyan (3mer), purple (6mer), yellow (9mer), and pink (12mer), respectively. The synthetic collagen peptide is colored green (PDB: 2F6A). The CNA protein is colored gray (PDB: 2F6A)

Based on the chitosan-collagen docking models previously described, the CNA structure was put back to the coordinates to check steric hindrance. In all the best docking results, the CNA N1 domain clashes to the chitosan with 3mer, 6mer, 9mer, or 12mer, from slightly to severely (Fig. 5). The residue Tyr 233 and Ser 235 of CNA mostly overlap with the 3mer chitosan, while Thr 221 slightly clashes (Fig. 5A). Chitosan polymers have the potential to prevent bacteria from binding to collagen peptides.

**Fig. 6:**
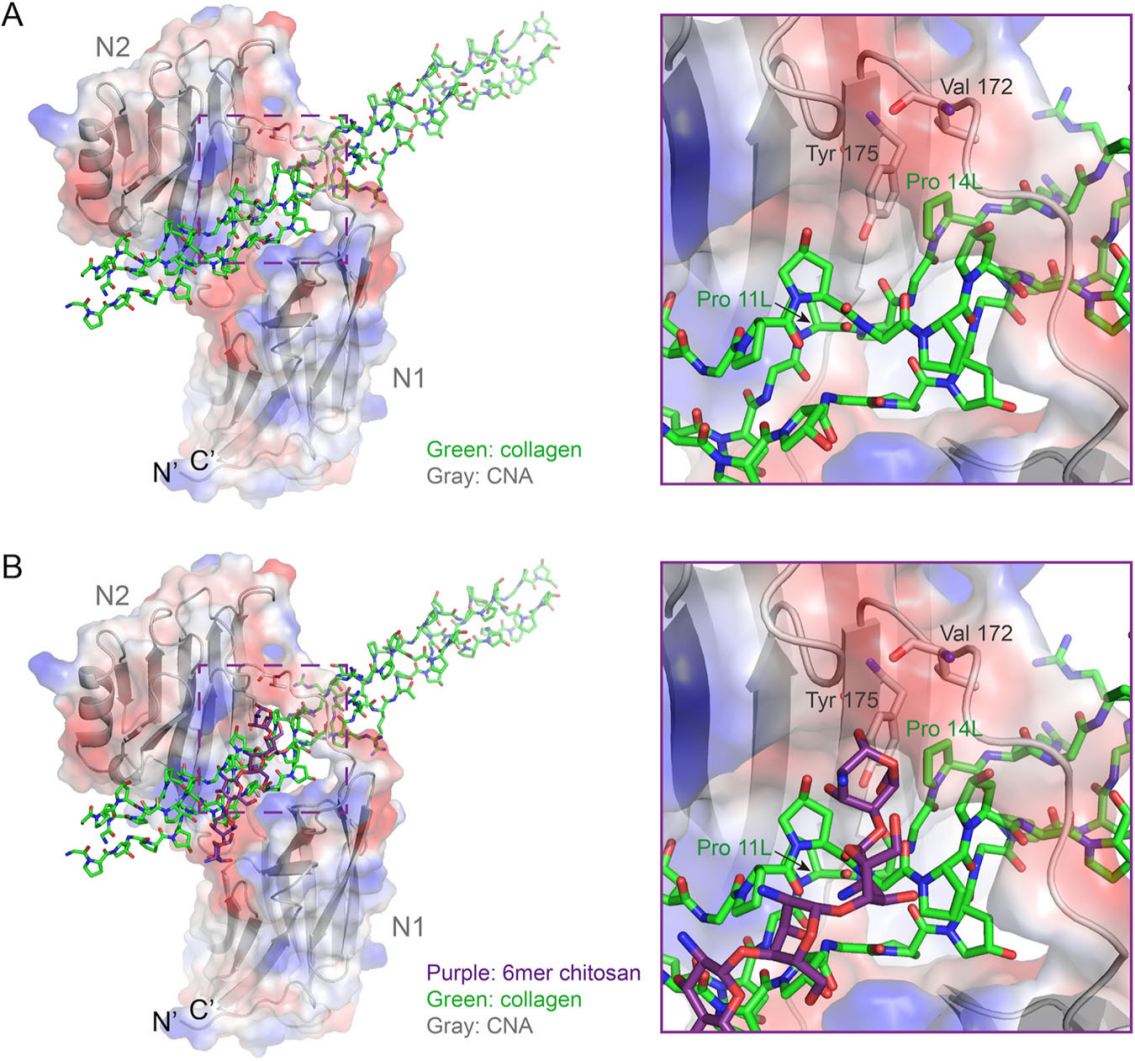
The best docking model of 6mer chitosan (**B**) disrupts hydrophobic interactions between collagen peptide and CNA protein (**A**). Semitransparency electrostatic potential mapped onto the CNA protein surface. Red denotes a negative charge (−52.7 kT/e), and blue denotes a net positive charge (+52.7 kT/e)

Moreover, the Val 172 residue from the CNA linker and Tyr 175 from the CNA N2 domain sandwich Pro 11 L from the collagen leading chain (Fig. 6A) [20]. The chitosan-6mer binding can disrupt such hydrophobic interactions by heading a positive charge group into the hydrophobic triangle area (Fig. 6B).

## Discussion

In this study, we demonstrated that the pretreatments to the root canal dentin affected Csnp distribution. Without UA, Csnp was aggregated and scattered sparsely on the dentin surface, while Csnp under UA was incorporated densely and evenly onto the exposed collagen type I (Fig. 1). More nanoparticles penetrated the deeper layer because UA thoroughly moistened the demineralized dentin surface and increased the adequate contact time and contact frequency between Csnp and collagen, providing more opportunities for the Csnp-collagen interaction. Moreover, UA also helped the Csnp molecules to scatter as much as possible in the solution, giving Csnp better penetration and distribution properties to the dentin. The Csnp-collagen combination brought a series of property changes to the dentin bonding interface, such as improving the mechanical strength and enzymatic resistance of dentin collagen fibers, giving the bonding interface the ability to inhibit bacteria, preserving demineralized dentin collagen fibers, and thus stabilizing the bonding interface [16].

The CFU values among groups were significantly different in our CFU assay, with the lowest CFU value in group B (*P* < 0.05, Table 1). The antibacterial effect of Csnp under UA was greater than that of Csnp in static immersion at the same concentration. The antibacterial efficacy was likely to depend on the amount and distribution of Csnp bound to the dentin surface. This observation was in agreement with the previous finding that Csnp exhibited outstanding antibacterial properties against *E. faecalis*-related dental pulp infection. The rate of bacterial killing by the Csnp depended on the concentration and time of interaction, and the antibacterial properties could be retained after aging for 90 days [29]. Csnp incorporated with carboxymethyl chitosan could disinfect root canal dentin and inhibit bacterial adhesion [30]. The action mode of cationic antibacterial agents was widely believed to interact with the cell envelope and then disrupt it. Raafat et al. showed that the electrostatic interaction with the teichoic acid of negatively charged Gram-positive bacteria was most likely associated with the primary contact between polycationic chitosan macromolecule and the bacteria [31, 32]. Charge interference reduced the antibiotic efficacy of chitosan on *E. faecalis* by affecting the electrostatic interaction between amino groups and cell membrane, and the cell envelope charge was confirmed to be associated with the antimicrobial activity of chitosan [13]. However, the nature of the components involved in the interaction interface with chitosan was rarely discussed.

**Table 1:** CFU of Csnp-pretreated dentin surfaces against *E. faecalis*

	A Control	B Csnp (UA)	C Csnp (static immersion)
CFU/ml	5.76 × 10^5 a^	8.13 × 10^3 b^	9.10 × 10^4 b^
LogCFU/ml	5.64 (0.5)^a^	3.49 (0.78)^b^	4.60 (0.69)^c^

Notes: different superscript letters indicate the statistical difference between the experimental groups (*P* < 0.05)

The Csnp-collagen connection and the bacterial resistance have a molecular structure basis. According to the docking results and functional experiments above, we found that the interactions of Csnp and collagen are naturally objective, imitating the interaction of the Csnp oligomers and dental collagen (Fig. 1). Although the computed affinities were not so strong, only raised from 2.28 mM to 590 μM (Fig. 2), we could still capture the opportunity that the steric closure between Csnp and collagen molecules. These modeling results were consistent with the SEM observation previously described, that Csnp was distributed more at the collagen exposed dentin sites (Fig. 1A), indicating the Csnp-collagen interaction on the dental surface. Using the EDC-crosslinking method, we turned the interaction of Csnp and collagen into covalent contact, setting them as an integral molecule. This pretreatment with the crosslinking agent could lead to a notable increase of the Csnp on the dentin surface, which was evenly distributed in the canal wall and dentinal tubules, especially in the areas with collagen partially exposed (Fig. 1B). Of course, raising the affinities of Csnp and collagen interactions high enough is a fantastic idea that Csnp may protect collagen without crosslinking for more convenient application, which is our next step to engineering the Csnp molecule with the help of the docking models.

The mechanism of the Csnp protection to collagen is the utilization of the inter-molecule steric effect, revealing the Csnp antibacterial property. The crosslinking treatments of collagen surface ensure Csnp binding efficiency and physical separation from bacterial collagen adhesin. In our Csnp-collagen docking models, Csnp occupied the grooves on the collagen surface and enhanced the structure intensity of the collagen triple helix, improving the mechanical strength of dentin collagen fibers (Figs. 2 and 3). The Csnp-collagen interaction mode showed that collagen was in the center axis with Csnp surrounding outside. Csnp enwrapped the collagen molecule, physically separating the collagen from the environment, not only the bacterial collagen adhesin (Fig. 5). This could be the structural basis of Csnp to give the bonding interface the ability to inhibit bacteria, improve enzymatic resistance of dentin collagen fibers, preserve demineralized dentin collagen fibers, and thus stabilize the bonding interface. As the molecular weight of chitosan increases, the volume of steric clash also gradually increases, while the binding affinity decrease (Fig. 5). We hypothesize an optimal length of the Csnp with the best collagen-CNA binding disturbance and suitable binding affinity for EDC-crosslink initiation.

These docking models have some limitations. The lengths of the Csnp molecules were only set to 12mer as the longest because the computations would massively increase while the polymer extended. Therefore, the models were close to the low molecular weight Csnp, especially to that dispersing well in solution, like the situation of Csnp treatment with ultrasonic agitation in the SEM observation. In addition, we used the crystal structure of *S. aureus* CNA in complex with the collagen peptide (PDB: 2F6A) instead of *E. faecalis* ACE, because of their similar structures [28] and the lacking of the ACE-collagen complex structure in the database. Nevertheless, our docking models explained the mode of Csnp-collagen molecular interaction and the Csnp anti-adhesive protection mechanism for dentin against collagen-binding bacteria, laying a solid foundation for future studies.

## Conclusion

Csnp protects dentin against *E. faecalis* by interacting and crosslinking with collagen type I by occupying the grooves of the collagen surface and preventing bacterial collagen adhesin binding through steric hindrance effect and interrupting hydrophobic interaction.

## Data Availability

The data that support the findings of this study are available from the corresponding author upon reasonable request.
